# Transection of Duplex Ureter During Vaginal Hysterectomy

**DOI:** 10.7759/cureus.6597

**Published:** 2020-01-08

**Authors:** Amenda Ann Davis

**Affiliations:** 1 Obstetrics and Gynecology, All India Institute of Medical Sciences, New Delhi, IND

**Keywords:** ureter injury, embryology, hysterectomy, congenital anomalies, surgical complications, urogynecology

## Abstract

Duplex ureter, an embryological developmental anomaly, can lead to intra-operative injuries, even by surgeons with a stronghold on normal ureteric anatomy. We describe the first case of an ectopic ureter transected during vaginal hysterectomy performed for pelvic organ proplase, due to its abnormally low implantation into the bladder, worsened by cystocoele. The injury was recognised, and the duplex ureter was diagnosed with cystoscopy and retrograde pyelography. A post-operative computed tomography urogram allowed us to map the exact course. In this case, there was injury to the ectopic, non-functional ureter, thus averting any further intervention. However, lower urinary tract injuries are serious complications with high morbidity, especially during delayed diagnosis. Knowledge of the ureter variants, meticulous tracing of the course, and use of post-operative cystoscopy could reduce these complications, particularly in face of increasing minimally invasive approaches.

## Introduction

Iatrogenic ureteric injury is a significant intra-operative concern for gynaecologists, owing to its proximity to the uterine arteries and cervix, retroperitoneal pelvic course, and at the infundibulopelvic ligament [1]. The overall incidence of iatrogenic ureteric injuries during gynaecological surgeries is less than 1%-2%, and even less during vaginal hysterectomy [2]. Normal ureteric anatomy is ingrained with due diligence, but unanticipated ureteric abnormalities may lead to intra-operative injury. Duplex ureter is estimated to occur in one in 500 people, and reported cases of injury during hysterectomy are rare. With increasing rates of minimally invasive hysterectomy, a known risk factor for ureter injury, it is essential to revisit embryology and understand the various developmental anomalies that may be encountered more frequently.

## Case presentation

A 65-year-old woman, post-menopausal since 20 years, had the complaint of a mass descending through the vagina since the past 30 years, which had gradually worsened with time. She also complained of stress urinary incontinence (SUI) and increased frequency of micturition since three years and had taken multiple courses of antibiotic therapy. The mass required manual reduction and splinting during micturition. She had no history of vaginal discharge or post-menopausal bleeding. She had five term vaginal deliveries which were uneventful. She had no history of chronic cough, constipation, or heavy weight lifting.

On examination, she was found to have third degree cervical descent, 3+ cystocoele, 1+ rectocoele, no enterocoele, and a keratinised area of congestion over the posterior lip of the cervix, POPQ (pelvic organ prolapse quantification) stage III with cervix as the leading point [3]. Stress test for SUI and Bonney’s test were positive. She had been advised pelvic floor training exercises many years ago, which led to a minimal improvement of symptoms, but due to worsening of the prolapse and urinary symptoms, a surgical plan for vaginal hysterectomy and pelvic floor repair was made, with pre-operative vaginal packing with acriflavine-glycerol for two weeks. Her pre-operative evaluation included a Papanicolaou smear, cervical biopsy, urine analysis, and ultrasound of abdomen and pelvis (Table 1).

**Table 1:** Pre-operative Investigations

Pap smear	Negative for malignancy
Cervical biopsy (from keratinised area)	Superficial strips of hyperplastic stratified squamous epithelium with epidermisation. Subepithelium shows mild chronic inflammation. No dysplasia
Urine routine microscopy	0-1 white blood cells/high-power field, no red blood cells, no bacteria
Urine culture	Sterile
Ultrasound abdomen and pelvis	Upper abdomen normal. Bilateral kidneys normal size, echoes, and corticomedullary differentiation. Uterus post-menopausal size, low lying, bilateral ovaries atrophic

Vaginal hysterectomy with pelvic floor repair was planned. The procedure began uneventfully, and the hysterectomy specimen was successfully removed. It was at the initiation of anterior colporrhaphy (cystocoele repair) that a steady drainage of purulent material was observed through the open vault, the origin of which was found to be a cylindrical structure at the vault margin. Upon passing a paediatric Foley’s catheter through it, 20 mL of pus was drained.

**Figure 1:**
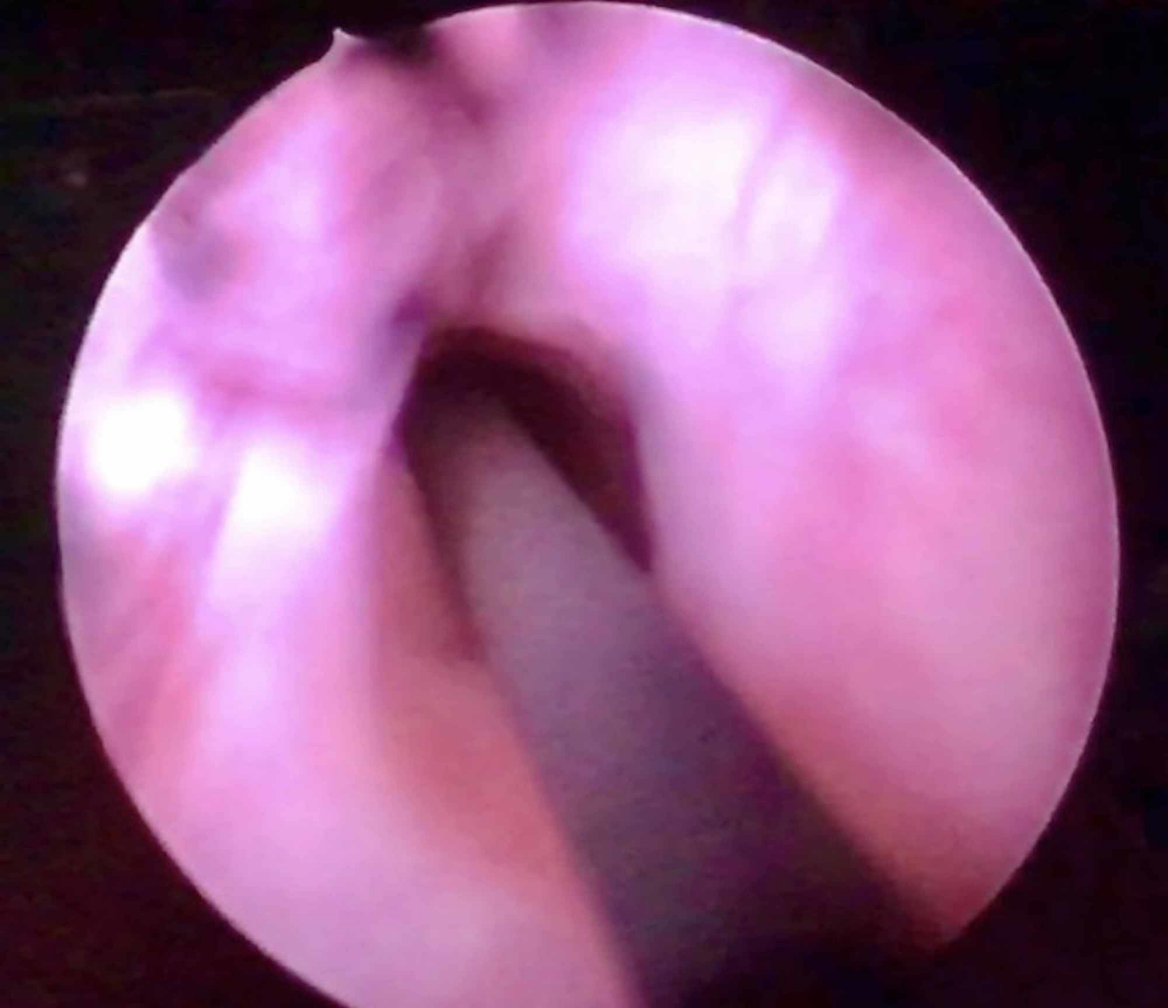
Cystoscopic view of the left ectopic ureteric orifice, inserting inferomedially into the bladder. The opening is dilated, with no efflux of urine. Passage of a guide wire led into the pelvis rather than the kidney, which was visualised by fluoroscopy.

Cystoscopy and retrograde pyelography were performed to delineate and confirm the suspected ureteric transection. On cystoscopy, the right ureteric opening was seen normally, with normal efflux. Two left ureteric openings were seen. A guide wire inserted through the inferior opening just proximal to the external sphincter seemed to pass into the pelvic cavity and not the kidney, and no efflux was visible from this orifice (Figure 1).

**Figure 2:**
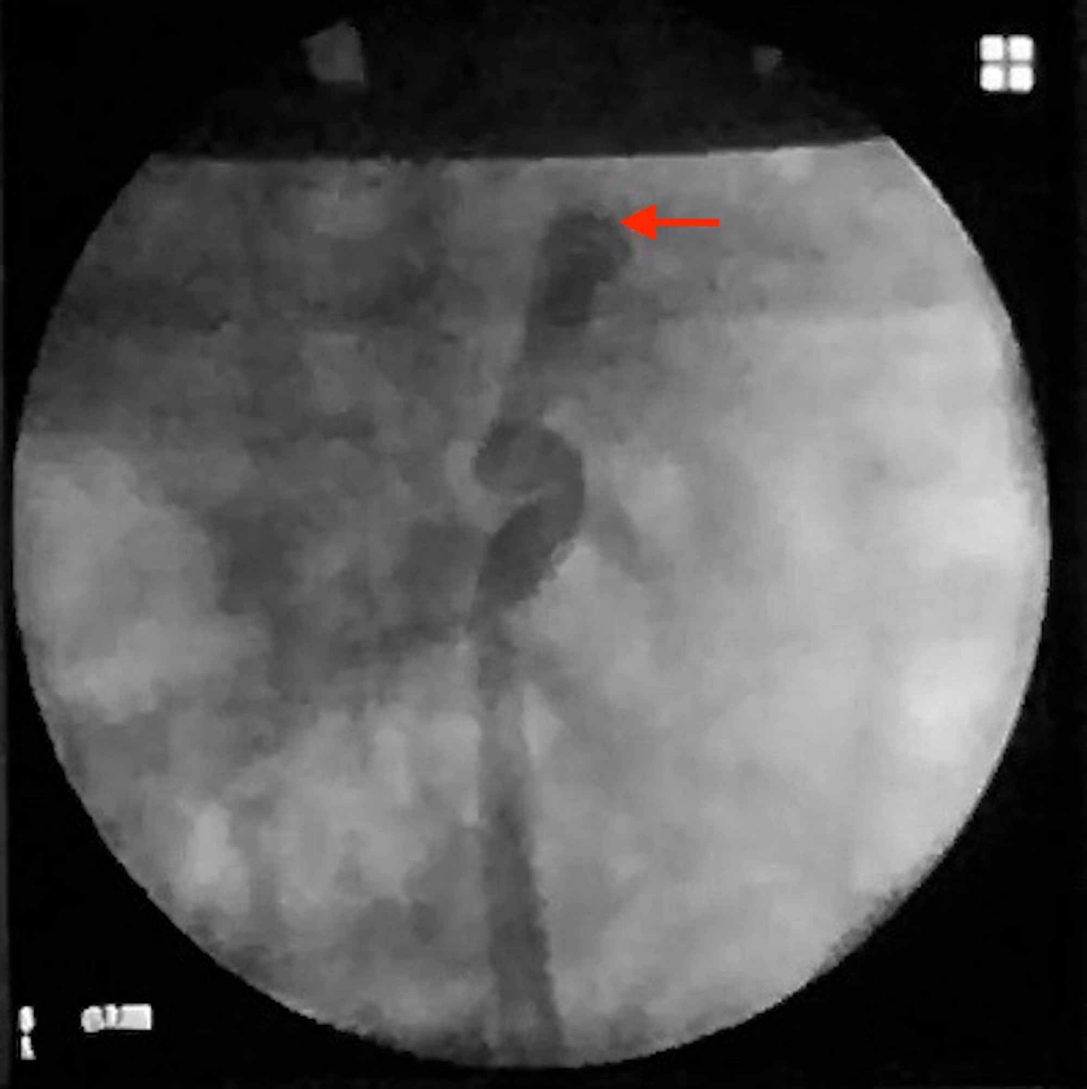
Retrograde pyelography performed via the cut end of the ureter in the vault, demonstrating a blind end (red arrow), with no connection to the pelvicalyceal system.

Bladder capacity appeared normal. Retrograde pyelography was done from the cut end of the ureter which delineated an apparently blind proximal end, with no contrast in the pelvicalyceal system (Figure 2).

**Figure 3:**
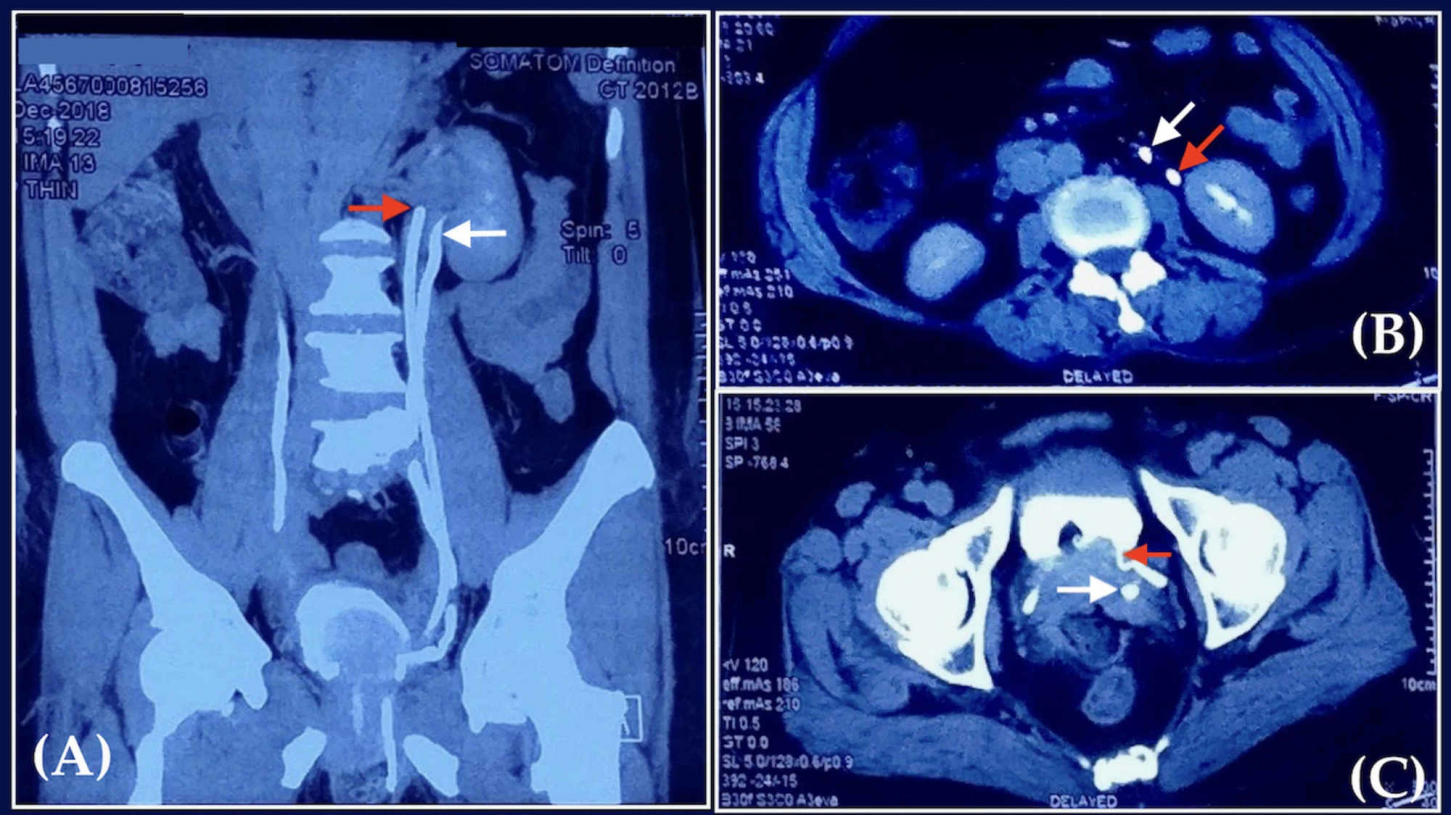
Post-operative computed tomography urogram (A) Coronal view showing the presence of two left ureters (red and white arrow). (B) Axial view showing orthotopic (red arrow) and ectopic (white arrow) left ureters in proximity to the left kidney. (C) Axial view at the level of bladder, showing the insertion of orthotopic ureter (red arrow) into the superior-lateral aspect. The transected ectopic ureter (white arrow) is not attached to the bladder.

A ureteric access catheter was placed in the cut end of the presumed left ectopic ureter and fixed. The vault was closed; anterior colporrhaphy with Kelly’s stitch and posterior colpoperineorrhaphy were completed. The patient had an uneventful post-operative period. A contrast computed tomography (CT) urogram was obtained on the second post-operative day to confirm and map the ureteric system (Figure 3).

**Figure 4:**
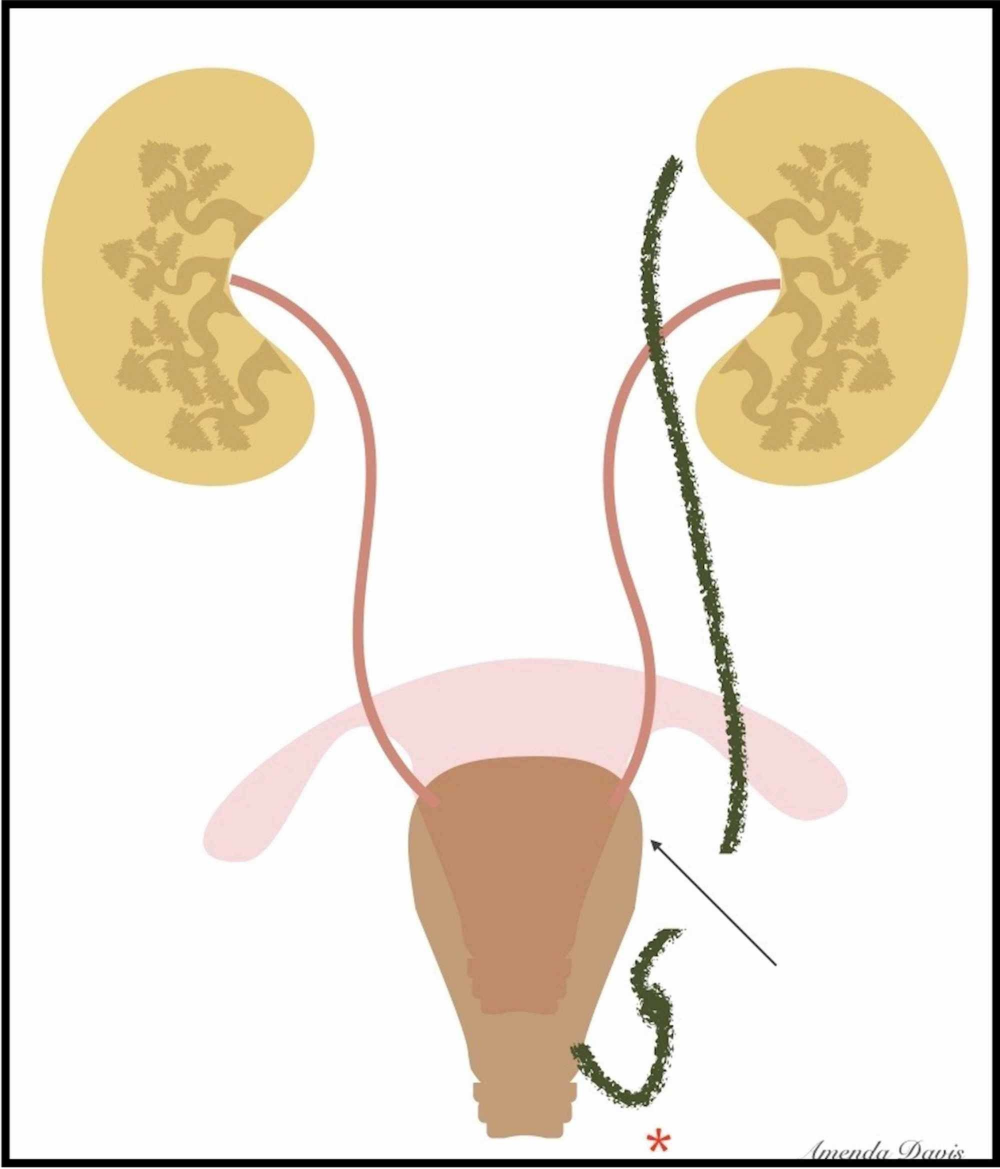
Illustration of the mapped ureteric system and the level of transection. The ectopic ureter (green) may have been kinked or pulled down due to its inferior insertion or prolapse (red asterisk), contributing to its injury.

We concluded that the upper kidney pole and the ectopic ureter (which was transected) were rudimentary and no active intervention was necessary (Figure 4).

The patient was discharged with urethral catheter on post-operative day 4 to promote epithelialisation of the ectopic ureter which was inserting into the bladder. She was doing well on follow-up at two weeks, and the catheter was removed. There were no residual or new urinary symptoms. 

## Discussion

This case demonstrates the potential risk of iatrogenic injury to anomalously located ureters. While we identified the injury intra-operatively and happened to transect the non-functioning ureter, there is always the possibility of damaging the functional ureter and delay in diagnosis. The need for laparotomy was averted in our case as we correctly assumed the rudimentary status of the cut ureter; nevertheless, it is often required. The presence of pyoureter, which led to recognition of injury, may have been due to vesicoureteric reflux and chronic infection, which was worsened by pelvic organ prolapse. The transection of this blind-ending ureter may theoretically reduce the subsequent incidence of urinary tract infection (UTI).

Duplication of ureter is one of the most common renal developmental anomalies, occurring in one in 500 people, and may be complete or incomplete [4]. On the 28th embryological day, the ureteric bud grows into the metanephros, leading to mutual induction of the other. If there are two ureteric buds, the result is complete duplication. Disruption or fissure of the ureteric bud leads to incomplete duplication or a blind-ending bifid ureter [5]. Ureteric duplication covers a spectrum, with the ectopic ureter inserting into various parts of the orthotopic ureter or the bladder. According to the Weigert-Meyer law, the ureter whose orifice is inferiomedial (ectopic ureter) arises cranially [6]. This described case is complete duplication with a proximally blind-ending ectopic ureter inserting into the lower part of the bladder. Such cases are usually clinically insignificant and lead to recurrent UTI and vague abdominal pain.

This case is, to the best of our knowledge, the first to describe an intra-operatively recognised injury to an ectopic ureter during vaginal hysterectomy. Dalzell et al. had described a case of duplex ureter damaged during laparoscopic hysterectomy which lead to ureterovaginal fistula six weeks after surgery [7]. Notably, in this case, the patient’s duplex ureter was not diagnosed pre-operatively even with a CT scan. Ergani et al. described a woman who presented with urinoma 20 days after abdominal hysterectomy due to injury to an anomalously located duplex ureter, necessitating ureteroureterostomy [8]. Of all the routes of hysterectomy, the risk of ureteric injuries, in general, is found to be maximum with laparoscopic route and minimal with vaginal route, perhaps due to the continuous downward traction on the cervix [9]. The latter, however, in our case, may have pulled the ectopic ureter as well. Certain society recommendations strongly advise routine post-operative cystoscopy after laparoscopic hysterectomy, as it can detect nearly 80%-90% of all injuries and significantly reduce the morbidity of delayed diagnosis [9].

## Conclusions

Duplicated ureteric systems may cause unanticipated intra-operative injuries. Intra-operative cystoscopy is an excellent way to diagnose lower urinary tract injury, whether used selectively or universally. The mapping of a complicated ureteric system may require multiple diagnostic modalities like cystoscopy, retrograde pyelography, and CT urogram.
